# Impact of Drying on Phytonutritional Compounds, In Vitro Antioxidant Activity and Cytotoxicity of Spiny Saltbush (*Rhagodia spinescens*)

**DOI:** 10.3390/antiox13111382

**Published:** 2024-11-12

**Authors:** Sephora Mutombo Mianda, Jiaxuan Li, Saleha Akter, Oladipupo Adiamo, Dharini Sivakumar, Yasmina Sultanbawa

**Affiliations:** 1 Phytochemical Food Network, Department of Crop Sciences, Tshwane University of Technology, Pretoria 0001, South Africa; miandamutombos@tut.ac.za; 2 Centre for Nutrition & Food Sciences, Queensland Alliance for Agriculture and Food Innovation, The University of Queensland, Brisbane, QLD 4068, Australia; jiaxuan.li2@uq.net.au (J.L.); saleha.akter@uq.edu.au (S.A.); o.adiamo@uq.edu.au (O.A.); y.sultanbawa@uq.edu.au (Y.S.)

**Keywords:** halophyte, salt alternatives, antioxidants carotenoids, phenolics, nutritional compounds

## Abstract

The Spiny saltbush (*Rhagodia spinscens*) is a halophyte species with the potential to provide natural ingredients used in food and pharmaceutical industries. In food and pharmaceutical applications, drying is necessary to maintain shelf-life, which reduces phytonutrient content. In this study, changes in the nutritional composition, phenolic and carotenoid profiles of radical antioxidant scavenging activity [(2,2′-azino-bis(3-ethylbenzothiazoline-6-sulfonic acid)(ABTS)], antioxidant power [ferric reducing antioxidant ability assay (FRAP)], and cytotoxicity of freeze- and oven-dried (55 °C for 24 h) spiny saltbush were determined. Sodium (4.72 g/100 g dry weight (DW), potassium (6.86 g/100 g DW), calcium (4.06 g/100 g DW), zinc (372 mg/kg DW) and protein content were higher in oven-dried samples than freeze-dried samples. Ultra-performance liquid chromatography–mass spectrometry analysis detected 18 metabolites in saltbush extracts. Partial Least-Squares Discriminant Analysis, Hierarchical Cluster Analysis, and Variable Importance in Projection discriminated between freeze-dried and oven-dried samples. Freeze-dried samples retained more individual metabolites than oven-dried samples, while oven-dried samples had higher antioxidant activity (ABTS and FRAP), lutein, trans-β carotene, and cis-β-carotene. Correlation analysis identified potential antioxidant candidates between phenolic and carotenoid compounds. Neither freeze-dried nor oven-dried spiny saltbush samples showed cytotoxicity. The study uncovered changes in phytonutritional compounds after the oven and freeze-drying spiny saltbush, a potential salt alternative and functional ingredient for the food industry.

## 1. Introduction

Globally, salt affects over 833 million hectares of soil, which is 8.7% of the planet Earth. Africa, Asia, and Latin America are the most common places to find them in arid or semi-arid environments [1]. In addition, more than 1.5 billion individuals around the globe encounter major difficulties in cultivating crops because of soil degradation due to 20 to 50% of all irrigated soils being too salty. Sustainable Development Goals depend upon healthy soil for improved food production, better nutrition, a cleaner environment, and better health for all [1]. Halophytes are adapted to thrive in a saline environment of more than 200 mM NaCl [2]. Halophytes’ adaptation to salinity and drought is mediated by stress-induced biochemical mechanisms involving phenolics, alkaloids, polysaccharides, and lipids [3]. Approximately 45 million ha (20% of all farming land) are affected by salt; halophytes can help produce food on saline lands in the future [4].

Halophytes have adapted to high-salinity, high-temperature, and high-irradiance environments by producing antioxidant, osmoprotective, and photoprotective metabolites, making them potential sources for the future discovery of highly beneficial phytochemicals [5]. These secondary metabolites (phenolics and flavonoids) also have health-enhancing properties (antioxidants, anti-inflammatory), making them valuable for food, pharmaceuticals, and cosmetics. The sea buckthorn plant (*Hippophae rhamnoides* L.) is a source of dietary supplements and cosmetic ingredients [6]. Furthermore, quinoa (*Chenopodium quinoa* Willd) and sea asparagus (*Salicornia* sp.) were known as food [7]. Furthermore, these halophytes include sodium, potassium, calcium, magnesium, sodium, dietary fiber, vitamin C, and vitamin A [8].

*Chenopodium spinescent* (Amaranthaceae) is commonly known as the spiny saltbush and referred to as *Rhagodia spinescens.* Traditionally, this species has been used as a salty, leafy vegetable in Australian cuisine, and it can also be used as a salt substitute. However, comprehensive details about spiny saltbush’s nutritional composition and quality remain largely unexplored. Halophytes, a naturally occurring ingredient that has functional and beneficial health benefits, is an ideal product for the design of novel products in the food and pharmaceutical industry.

In saline lands, spiny saltbush is cultivated for commercial use in land restoration programs aimed at addressing salinity. It also has potential commercial value as a salt alternative and natural antioxidant. The spiny saltbush leaves are highly susceptible to decay, so freezing or drying them ensures a long shelf life, better quality, and better stability of phytochemicals while reducing browning enzyme activity and microbial growth [8]. Spiny saltbush must be marketed in a manner that benefits both the food and pharmaceutical industries to be exploited commercially. The purpose of this work was threefold: to explore the effects of drying methods (1) on the changes in the nutritional of fresh spiny saltbush, (2) phenolic and carotenoid modifications, followed by chemical characterization of extracts, (3) determination of radical scavenging activity and toxicological properties in vitro.

## 2. Materials and Methods

### 2.1. Chemicals

All solvents (HPLC and analytical grade) were bought from Thermo Fisher Scientific (Woolloongabba, QLD 4102, Australia). All other chemicals were purchased from ChemSupply Australia (Gillman, Adelaide, SA 5013, Australia).

### 2.2. Preparation of Raw Material

**Figure 1:**
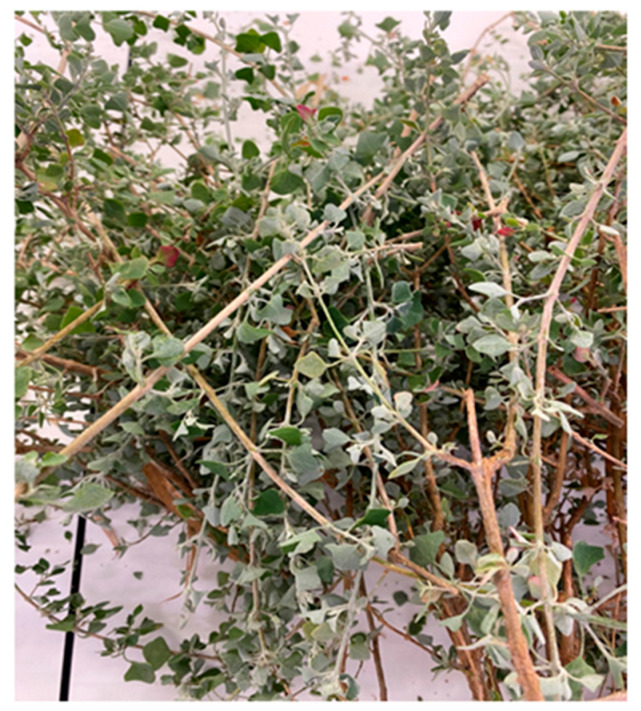
Spiny saltbush (*Rhagodia spinescens*).

The spiny saltbush (*Rhagodia spinescens*) samples (Figure 1) were collected in Brisbane in 2023 and transported to the laboratory within 2 h of harvest. A total of 8 kg of leaves were removed from the stalks, and 4 kg were dried at 55 °C in an oven (Steridium, DG-160) (Steridium, Brendale, QLD, Australia) for 24 h (The drying time and temperature were chosen according to industry recommendations.). The other 4 kg of leaves were frozen-dried using a benchtop freeze dryer (Free Zone^®^, 8 L, 50 °C) (Labconco, Kansas City, MO, USA), then for further analysis, they were pulverized into a fine powder with a hammer mill (KinematicaTM Polymix^TM^ PX-MFC 90 D, Thermo Fisher Scientific, Scoresby, VIC, Australia).

### 2.3. Color and Water Activity Assessments

The color parameters (L*, a*, b*) were determined using a colorimeter (Minolta CR-400 Chroma Meter) (Konica Minolta, Osaka, Japan) [9]. The samples’ water activity (Aw) was ascertained at 25 °C with a LabTouch-aw water-activity meter (Novasina AG, Lachen, Switzerland) according to Yu et al. [10]. Each measurement was conducted three times.

### 2.4. Proximate Analysis

Ash, moisture content, crude protein, fat, and total dietary fiber were determined based on the AOAC method #923.03, AOAC method #925.10, method #990.03, AOAC #960.39, AOAC Method #991.43, respectively. Minerals and trace elements were determined using coupled plasma optical emission spectrometry (ICP-OES) (Symbio Alliance Laboratories Pty Ltd. Brisbane, QLD, Australia) (an accredited National Association of Testing Authorities, NATA, laboratory) using AOAC methodology [11].

### 2.5. UPLC-MS Profiling of Metabolites and Targeted Phenolics

The metabolite profiling of spiny saltbush was conducted following the method described by Tshilongo et al. [12] without any modification. Powdered samples (2 g) were extracted with 1% formic acid in 50% methanol (15 mL) with sonication in a sonication bath (Skymen ultrasonic, model: JP-100S, Shenzhen, China) for 1 h before centrifugation. Then, the liquid extract was collected and further diluted 10 times with 0.1% formic acid (FA) in 50% methanol. Diluted extracts (0.5 µL) were injected in a Waters Acquity UPLC (Waters, Milford, MA, USA) equipped with a Waters qTOF mass spectrometer and a PDA detector. A Waters HSS T3 column (2.1 mm × 150 mm, 1.7 μm) maintained at 60 °C was used for compounds’ separation, with a gradient elution of 0.1% FA (solvent A) and acetonitrile + 0.1% FA (solvent B). Data were processed with MSDIAL and MSFINDER. Potential compounds were identified from databases.

### 2.6. Carotenoids Analysis

Carotenoids were extracted according to Djuikwo et al. [13]. The samples were homogenized for 10 min in 95% ethanol containing 0.1% butylated hydroxytoluene (BHT). Saponification was performed in methanol with 20% KOH solution for 30 min continuously shaking. Following that, hexane-dichloromethane (70:30, *v*/*v*) with 0.1% BHT was added. A 10% NaCl solution (*w*/*v*) was inserted to facilitate phase separation, then centrifuged at 3900× *g* for 5 min. After collecting the hexane phase, it was evaporated, and the extract was redissolved in a methanol/MTBE mixture (50:50, *v*/*v*) with 0.1% BHT. For total carotenoid content (TCC), the extract absorbance was read at 450 nm using a spectrophotometer. TCC was calculated using a standard curve generated with β-carotene and expressed as β-carotene equivalent in mg/100 g.

The carotenoid extract (10 µL) was injected into the Shimadzu HPLC instrument (LC-2030C 3D, Kyoto, Japan) for identification and quantification of individual carotenoids as described by Bhengu et al. [14] without any modification. Chromatograms were monitored at 460 nm. External standards were used for quantification.

### 2.7. Extraction of Phenolic Compounds

Extraction of phenolics from the dry spiny saltbush was performed according to Hong et al. [15] using 0.5 g of dry powder mixed with 5 mL of 80% aqueous acidified (0.1%) methanol. The resulting solution was Vortexed (RATEK shaker RATEK, Boronia, VIC, Australia) for 30 s and then shaken at 200 rpm for 15 min. The supernatant was collected, filtered, and then kept at −35 °C for antioxidant activity determination.

### 2.8. Ferric-Reducing Antioxidant Power Assay

FRAP value was ascertained as depicted by Phahlane et al. [16] with no changes. In a microplate reader, 240 µL of FRAP reagent was mixed with 10 µL of leaf extract (2.5.4) within 1–2 h. In darkness, the mixture was left to react for 5 min before readings were taken with a Varioskan Lux Microplate reader (Thermo Fisher Scientific, Singapore). The results were determined using the calibration curve with the Trolox standard and presented as TEAC mM/g dw.

### 2.9. 2,2-Azino-Bis-3-Ethylbenzothiazoline-6-Sulfonic (ABTS) Scavenging Activity

ABTS assays were conducted according to Xiao et al. [17]. The working solution was made by mixing 5 mL of 7 mmol/L ABTS solution in acetic acid buffer (pH 4.6) with 5 mL of K_2_S_2_O_8_ solution (2.45 mmol/L), which was held at 25 C° for 16 h. The 11-fold diluted ABTS working solution with phosphate buffer was kept at 25° C for 30 min. Then, 200 µL of the diluted solution was mixed with the sample extract (Section 2.7) in 96 well plates. Using a Varioskan Lux Microplate reader (Thermo Fisher Scientific, Singapore), the reaction was determined at absorbance 743 nm after 7 min using Trolox as a standard. The results expressed as µmol Trolox equivalent (TEAC)/g DW was calculated against the Trolox calibration curve.

### 2.10. Cytotoxicity Analysis

Cytotoxicity analysis was conducted according to Akter et al. [18]. Two intestinal cell lines, Caco2 and HT29-MTX0-E12 were purchased from Sigma-Aldrich (Sydney, NSW, Australia). Cells were cultured at 37 °C with 5% CO_2_ in DMEM supplemented with 10% FBS. Cells were subcultured upon reaching around 80% confluency and monitored using an EVOS M5000 imaging system (Thermo Fisher Scientific, Singapore). Cells were washed with DPBS and detached using 3.5 mL of 0.25% trypsin-EDTA for about 4–5 min at 37 °C. Microscopic examination confirmed detachment, and the reaction was neutralized with DMEM containing 10% FBS. After centrifuging at 1400× *g* for 5 min, the cell pellet was resuspended in fresh medium and transferred to new flasks at ratios ranging from 1:2 to 1:6 to promote optimal growth. All procedures were performed in a sanitized biosafety cabinet. The passage numbers for both cell lines were between 11 and 25 used for the assays. Caco2 and HT29-MTX-E12 cells at a density of 4 × 10^4^ Cells/well were grown in 96 well plates for 24 h prior to treatment, producing 90% cell confluency. The assay uses Nunc F96 MicroWell Black polystyrene plates (Thermo Fisher Scientific (Woolloongabba, QLD 4102, Australia) where 100 μL of mammalian cell suspensions are added per well. The 80% aqueous methanol and water extracts of the freeze-dried and oven-dried spiny saltbush powder samples were prepared according to the protocol described in Section 2.7. The obtained extracts were concentrated using a Savant SpeedVac SPD300DDA vacuum concentrator (Thermo Fisher Scientific, Singapore). The concentrated water extract was again freeze-dried to obtain dry pellets, and then both the dry methanol and water extracts were reconstituted in DMSO and sterile ultrapure water respectively to use for the cell culture assays. The sample ‘SOD’ indicates ‘Saltbush oven-dried’, and ‘SFD’ refers to ‘Saltbush freeze-dried’. All the reconstituted samples were further diluted using HBSS and six different concentrations were used (in a logarithmic scale) to produce 0–100% cell viability and calculation of half-maximal inhibitory concentration (IC_50_). The concentration range for SOD methanol was 65355–0.65 µg/mL, SOD water 70563–0.70 µg/mL, SFD methanol 46620–0.46 µg/mL and SFD water 76557–0.76 µg/mL. The CyQUANT^®^ NF Cell proliferation assay was utilized for cell viability analysis [18]. The protocol involved multiple incubation and washing stages, with final fluorescence readings obtained using a VarioSkan LUX microplate reader at 485 nm excitation and 530 nm emission.

### 2.11. Statistical Analysis

The results of all biochemical analyses are presented as mean ± standard deviation (SD). GraphPad Prism v10 (GraphPad Software, Boston, MA, USA) was used to calculate IC_50_ values and display dose-response curves for percentage cell viability. The mean values of all experiments checked for significant differences between samples by performing a T-test, and *p*-values of 0.05 or less were considered statistically significant.

## 3. Results and Discussions

Table 1 presents the moisture content, trace elements, proximate content, and color of fresh spiny saltbush leaves.

### 3.1. Effect of Drying Methods on Moisture Content and Color Property

The fresh spiny saltbush showed a leaf moisture content of 87.19%, (Supplementary Table S1), which is very high, making the leaves prone to microbial growth, reducing shelf life, and leading to undesirable biochemical changes [19]. The color attributes measured on the fresh leaf samples are shown in Table S1. Spiny saltbush fresh leaves had the following color attributes: L* = 47.86, a* = −8.87 and b* = 13.17. The L* value indicates medium color brightness. The negative a* value signifies a prominent green component in the leaf color, while the positive b* value suggests the presence of a yellow component, though not very intense. Spiny saltbush leaves are dark grey-green when fresh. The color change value of freeze-dried (ΔE* = 14.93) and oven-dried (ΔE* = 12.63) samples are not significantly different (*p* > 0.05). But, the ΔE* values show that both dried samples are different from fresh samples. Extended exposure to cold temperatures (24 h) during freeze-drying might have influenced the colors. Additionally, drying in an oven at a gentler temperature (55 °C) helps to avoid further degradation of heat-sensitive color components like chlorophyll. It has been demonstrated that drying at 50 °C results in smaller quality loss when drying herbs and spices [5].

### 3.2. Effect of Drying Methods on Minerals, Trace Elements, and Proximate Composition

In recent years, dietary minerals have gained interest for their part in fighting a wide range of diseases. The key minerals, like magnesium, calcium, sodium, and potassium, are biologically essential and play important roles in metabolic processes in addition to maintaining water and acid-base balance. Furthermore, trace minerals (e.g., zinc and iron) significantly influence vitamins, hormones, and enzyme activities [20]. Ca, K, Na, Mg, Zn, and Fe were detected and quantified in spiny saltbush samples subjected to oven drying and freeze-drying (Table 1). Among these minerals, K, Na, and Ca were present in significantly higher concentrations. The results of this study are consistent with those of other research, indicating that Australian native edible halophytes are abundant in vital minerals and trace elements, especially calcium and potassium [4,21]. Oven-dried spiny saltbush had 4.72 g/100 g DW of Na concentration, whereas freeze-dried spiny saltbush contained 3.86 g/100 g DW of Na. Therefore, consuming 100 g of this dried spiny saltbush would result in a Na intake significantly exceeding international daily recommendations (0.46–1.3 g per day) [4]. Similarly, the K (6.86 g/100 g DW), Ca (4.06 g/100 g DW), and Zn (372 mg/kg DW) contents of oven-dried spiny saltbush were higher than that of freeze-dried samples (4.46 g/100 g DW, 2.83 g/100 g DW and 299 mg/kg DW, respectively). It may be that rapid evaporation of water at high temperatures during oven drying breaks down cellular structures more rapidly and releases more minerals than freeze-dried samples [20]. On the other hand, the minerals such as Na, K, Ca, and Zn increased during oven drying due to the removal of moisture, which consequently concentrated and released minerals [5]. Neither oven-dried nor freeze-dried samples differed significantly in Mg or Fe concentrations.

Table 1 shows the results, with oven-dried samples retaining significantly less moisture (3.44%) than freeze-dried samples (10.56%). As a result of the direct sublimation of water from ice crystals to steam, freeze-dry samples retained more moisture and had higher water activity [22]. As opposed to oven drying, which evaporates moisture using heat, oven drying results in the loss of more moisture. The physicochemical properties of processed foods depend on their moisture content. Lower moisture levels help minimize the risk of microbial growth, improve storage stability, and prevent unwanted biochemical changes [4]. In a similar study, Lim et al. [23] found that oven-dried pumpkin powder had lower moisture content and water activity than freeze-dried samples. Nonetheless, the oven and freeze-dried samples can both be considered safe concerning microbial growth because their moisture contents are lower than 15% and water activities <0.61 [5].

Freeze-dried samples retained significantly less protein (8.00%) than oven-dried samples (11.81%), whereas freezer-dried and oven-dried samples had no significant difference in fat content (3.97%). The drying method significantly (*p* < 0.05) affected the ash content and found that the freeze-dried sample had higher ash content 33.35%) than the oven-dried sample (28.79%) [24]. Oven-dried spiny salt bush has a lower ash content than freeze-dried leaves because the drying process can cause the loss of low boiling components and oxidation changes.

In our study, oven-dried and freeze-dried spiny saltbush had a higher ash matter than other edible plants. This suggests a high concentration of inorganic compounds in halophytes, which is attributed to their saline growing environments and their capacity to retain minerals [25]. Food nutrients can be enhanced or diminished depending on the drying method; freeze-drying is most effective for preserving plant nutrients and bioactive compounds over oven drying [26].

Oven-dried samples (54,110 mg/100 g) contained significantly more dietary fiber than freeze-dried samples (46,240 mg/100 g). In the oven, drying temperature affects the structural characteristics of the fiber, as well as variation in soluble dietary fiber content [27]. Similarly, when *Salicornia ramosissima* was oven-dried at 70 °C, its dietary fiber content increased an increase [28]. The impact of the drying temperature on dietary fiber suggests that low temperatures should be used when utilizing saltbush fiber as a food ingredient. Although Australia and New Zealand recommend an Adequate Intake (AI) of dietary fiber for adults, with men requiring 30 g/day and women 25 g/day [29], adding freeze-dried spiny saltbush powder to food can significantly improve dietary fiber intake, resulting in better gut health, and improved digestion [21].

### 3.3. Effect of Drying Methods on Untargeted Metabolite Profiles

**Figure 2:**
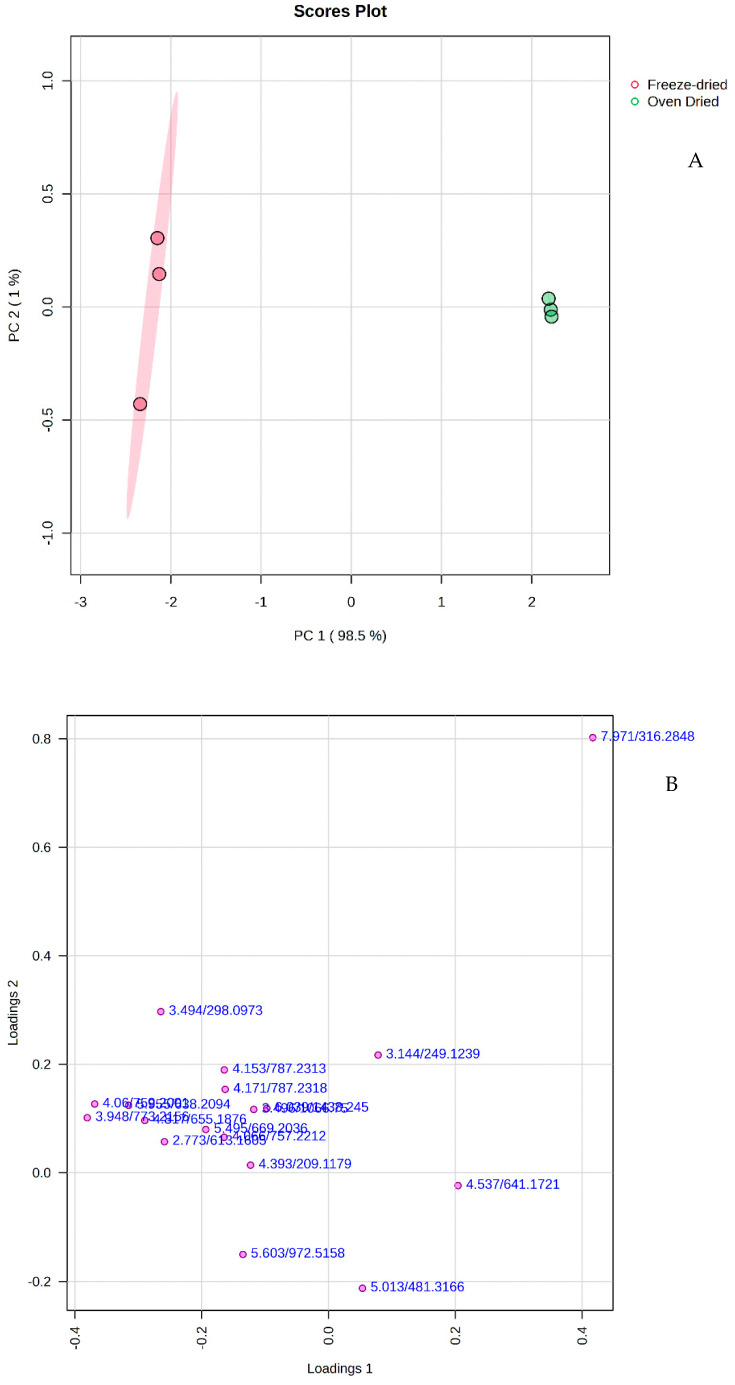
(**A**) An unsupervised PCA score plot of metabolites generated by UPLC-QTOF/MS analysis showing the separation of two clusters. (**B**) Loading of metabolites in the PCA score plot.

In widely targeted metabolomics, non-target metabolite detection methods work in combination with targeted metabolite detection approaches, culminating in enhanced robustness, coverage, and accuracy [30]. With the UPLC-MS analytical tool, the primary and secondary metabolites of spiny saltbush were tentatively identified after freeze-drying and hot air drying. Spiny saltbush metabolism can be linked to the phenomenon of upregulation of certain phytochemicals caused by increasing temperature by studying the metabolic profiles of plants dried in freezer dryers and hot air ovens. UPLC-MS analysis of saltbush extracts in 50% methanol revealed the relative abundance of 18 potential metabolites (Table S2, chromatograms in Figures S1–S9). These included eight flavonoid-glycosides, one nucleoside, two peptides, one thioglycoside, one hydroxyindole, one anisole, one amino alcohol, one ecdysteroid, and two unknown compounds. The level of similarities and differences, based on the relative intensities of the UPLC-MS untargeted metabolites, between the oven-dried and freeze-dried spiny saltbush was examined using unsupervised principal component analysis (PCA). The two-dimensional PCA score plot of PC1 versus PC2 (Figure 2A) explained 99.5% of the variance (98.5% and 1%, respectively).

**Figure 3:**
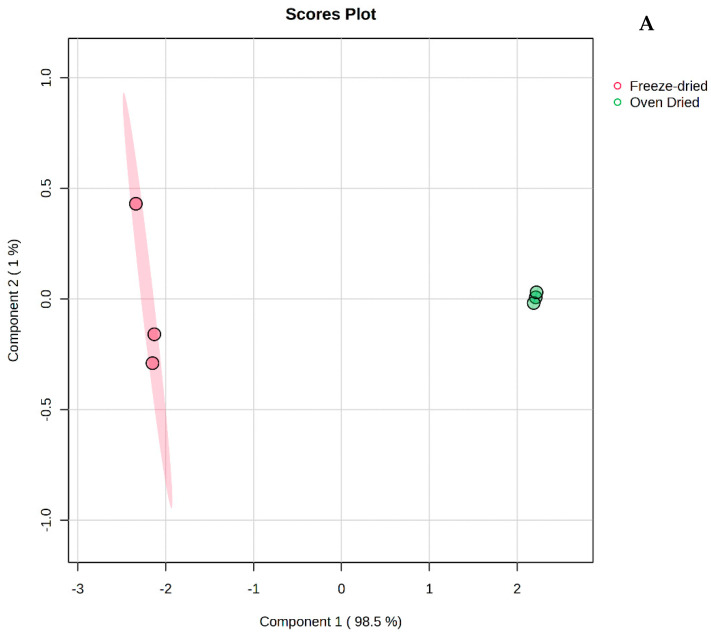

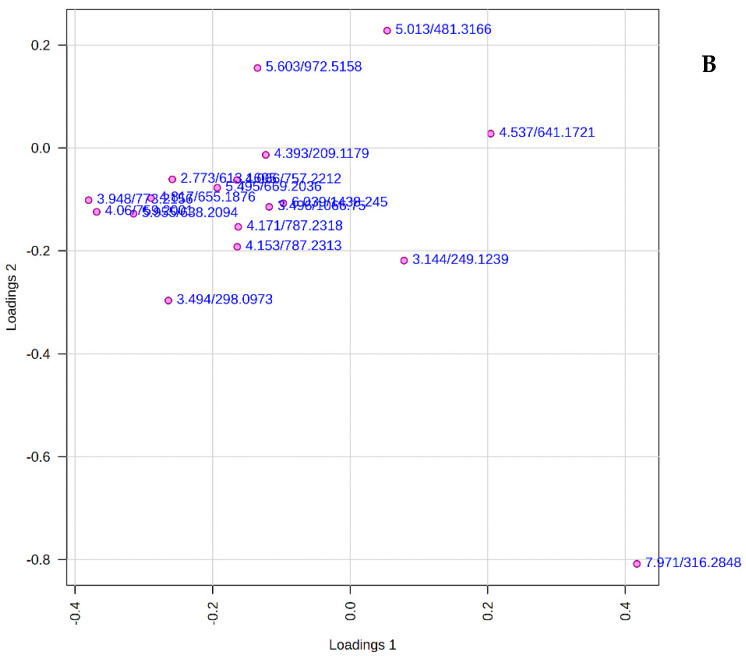
(**A**) A PLS-DA score plot showing the two drying methods separated into two groups. (**B**) PLS-DA score plots loaded with metabolites detected by UPLC-QTOF/MS.

Metabolites present in the oven-dried and freeze-dried spiny saltbush separated the two groups on the PCA score plot (Their corresponding loadings are given in Table S3). On the PCA loading plot (Figure 2B), the compound at *m/z* 316.284 (Retention time (RT): 7.971 min), identified as dehydrophytosphingosine, was loaded positively of PC1 (*r* = 0.41), while it was loaded positively on PC2 (*r* = 0.80). Further, PLS-DA was used to clarify the variation between the oven-dried and freeze-dried spiny saltbush based on their metabolite profiles. The first two components (PC1 98.5% and PC2 1%) explained 99.5% (Figure 3A) of the variation in the metabolites, which showed two clusters. Figure 3B displays the loading of the metabolites on the PLS-DA components 1 and 2, and the loading of metabolites is presented in Supplementary Table S4. The compound at *m*/*z* 316.2848 (dehydrophytosphingosine, RT:7.971 min) was loading positively on PC1 (*r* = 0.41), while the compounds at *m/z* 759.200 (RT: 4.06 min) and *m/z* 773.215 (RT: 3.948 min), identified as quercetin 3-sambubioside-7-glucoside and kaempferol 3-sophorotrioside, respectively, were loaded negatively (*r* = −0.37 and −0.38, respectively). On PC2, dehydrophytosphingosine was loaded negatively (*r* = −0.80). The PLS-DA model displays good prediction (*Q*^2^ = 0.99) and goodness-of-fit (*R*^2^ = 0.99) levels.

**Figure 4:**
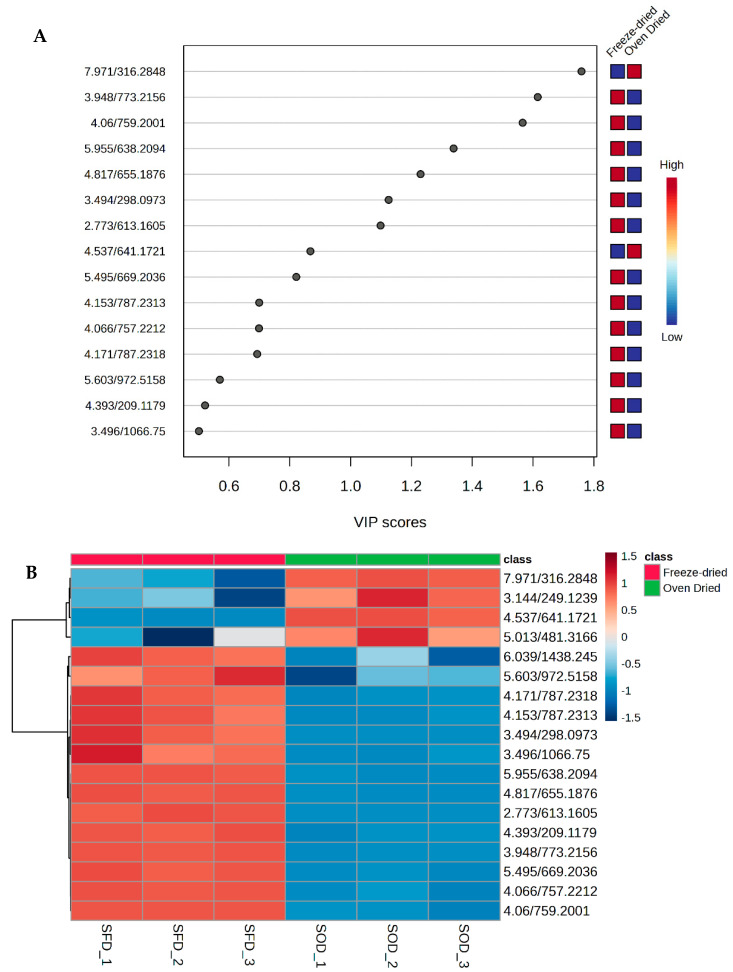
(**A**) VIP scores are allocated to metabolites in PLS-DA. The score they are assigned from low to high indicates the level of significance of variables. Each of the metabolites is represented by a colored box on the right. A high red level indicates a high level, while a low blue level indicates a low level. (**B**) Heat map. Colored areas on the map relate to abundances of bioactive metabolites. Each row corresponds to a bioactive metabolite, and each column denotes the type of drying treatment. Red represents high concentrations, and blue reflects low quantities.

The contribution of each metabolite to the separation of the groups was further evaluated by a variable importance in projection (VIP) score. The top compounds with a VIP score >1 were dehydrophytosphingosine (7.971/316.2848), kaempferol 3-sophorotrioside (3.948/773.2156), quercetin 3-sambubioside-7-glucoside (4.06/759.200), limocitrin 3-rutinoside (4.817/655.1876), 5′-methylthioadenosine (3.494/298.0973), and two unknown compounds (2.773/613.1605 and 5.955/638.2094), hence were considered to interpret the results. Except for dehydrophytosphingosine, all the top compounds were in higher concentrations in the freeze-dried samples (Figure 4A, Table S5). Furthermore, a heatmap was created based on metabolite concentrations. According to the heat map (Figure 4B), 14 out of the 18 detected metabolites were upregulated in the freeze-dried samples while four compounds were upregulated in the oven-dried saltbush. This indicated that freeze-drying preserved more compounds than oven-drying. It appears that physiological and biochemical reactions were triggered by oven drying. Different membrane lipids respond differently to abiotic stresses, and lipid-dependent signaling responses are induced. A recent study found that lipids act as signal transmitters in plants, decreasing stress tolerance and enhancing defences. Stress produces signaling lipids, such as sphingolipids like dehydrophytosphingosine [31], that could maintain membrane stability and heat tolerance. Ranupenin 3-rutinoside, the second metabolite, is a flavonoid-3-O-glycoside, which is a derivative of phenylpropanoid pathways induced under heat stress conditions [32]. Tryptophan, phenylalanine, and ornithine metabolism generate alkaloids like sespendole. Protein synthesis is regulated by amino acid metabolism, which serves as an intermediate for some metabolites and regulates multiple metabolic pathways [32]. A significant portion of these differential metabolites were derived from phenylpropanoid biosynthesis in freeze-dried samples. The frequency of phenylpropanoids and small peptides is higher after freeze-drying. Freeze-drying of samples increases metabolite concentrations by destructing cells and facilitating solvent extraction, improving extraction yields [33]. Contrarily, phenol concentrations in lemons decreased when subjected to low-temperature drying.

### 3.4. Effect of the Drying Methods on Targeted Phenolic Constituents

Table 2 presents the impact of the drying methods on individual phenols in spiny saltbush. A total of eight flavonoid-glycoside (kaempferol 3-sophorotrioside, quercetin 3-sambubioside-7-glucoside, kaempferol 3-gentiobioside 7-rhamnoside, isorhamnetin 3-O-[b-D-glucopyranosyl-(1->2)-[a-L-rhamnopyranosyl-(1->6)]-b-D-glucopyranoside], ranupenin 3-rutinoside, isorhamnetin-rhamnosyl-hexoside-glucoside, limocitrin 3-rutinoside, jaceidin 7-neohesperidoside) were identified and quantified in the freeze-dried and oven-dried spiny saltbush samples (Their MS/MS spectra are presented in Supplementary Figures S3–S9). There are no previous reports on the phenolic compounds of spiny saltbush. The results showed that all the phenolic compounds, except for ranupenin 3-rutinoside, were highly accumulated in the freeze-dried spiny saltbush as compared to the oven-dried samples. Similar to our results, Oliveira-Alves et al. [28] reported that compared to the freeze-dried halophyte plant *Salicornia ramosissima* J. Woods, the oven-drying process led to a 54% decrease in total flavonoids and a 20% decrease in total hydroxycinnamic acids. Freeze-drying effectively preserves the polyphenol compounds by preventing thermal and oxidative degradation, as well as minimizing enzymatic reactions catalyzed by polyphenol oxidase, lipoxygenase, and peroxidase [34]. Furthermore, kaempferol 3-sophorotrioside can be useful in treating inflammatory diseases of the blood vessels. Food and Drug Administration (FDA: National Drug Code numbers 65,448–3085, 65,448–3005) has approved quercetin as an anti-allergic and antioxidant ingredient [35]. Due to its positive health benefits, quercetin has become a highly sought-after nutraceutical ingredient. The health benefits of quercetin include antiviral, anti-inflammatory, antineoplastic, antioxidant, cardio-protective, anti-obesity, antitumor, and antibacterial properties [36]. The quercetin-based treatment of COVID-19 may provide an exciting prospect in light of the quercetin’s strong activity [37]. Biological activities of isorhamnetin glycosides against cancer, obesity, diabetes, thrombosis, and hepatitis have been demonstrated.

### 3.5. Effect of the Drying Methods on Carotenoids

Carotenoids are known for their positive health effects, including cancer prevention and provitamin A activity [36]. The carotenoid content of spiny saltbush samples is shown in Table 3. Carotenoids are susceptible to oxidation and heat-induced breakdown. Freeze-drying, with its cooler processing temperature and gentle drying process, effectively shields carotenoids from temperature-induced deterioration. The freeze-drying process preserves heat-sensitive carotenoids by freezing and sublimating them under vacuum [38]. Further, the results showed that trans-β carotene was unaffected by the drying method, while lutein, cis-β carotene and zeaxanthin were significantly higher in the oven-dried samples and zeaxanthin was not detected in the freeze-dried samples. Although there is an established Recommended Daily Intake (RDA) for vitamin A, there isn’t a specific RDA for beta-carotene. However, studies have explored dosages ranging from 15 to 180 mg per day [39]. Therefore, consuming 0.668 to 8 g of dried spiny saltbush daily would be adequate to achieve this intake. Likewise, there are no RDAs for lutein and zeaxanthin; nonetheless, 6 mg of lutein per day has been suggested by some researchers, which means that ingesting 12.2 g of freeze-dried and 9.30 g of oven-dried spiny saltbush would achieve this 6 mg intake of lutein (NIH). Zeaxanthin and lutein are the most prevalent carotenoids in optical tissues. In addition, their inherent anti-oxidant properties, manifested as anti-inflammatory properties, help prevent age-related diseases, such as macular degeneration and cataracts. According to our study, trans and cis-β-carotene levels did not vary throughout the drying process. In contrast, during oven drying, the amount of cis-carotene significantly increased. All-trans isomers have lower bioaccessibility and bioavailability since they tend to crystallize and cluster more readily than their cis isomers, which are typically shorter and simpler to disperse into micelles. The cis isomeric form increased after heating, and isomerization rates were associated with heat treatment intensity and duration [40,41]. Nevertheless, carotenoid components may increase during oven drying due to thermochemical reactions and pigment breakdown [42].

### 3.6. Effect of the Drying Methods on Antioxidant Activity Spiny Saltbush

The antioxidant activities of oven- and freeze-dried spiny saltbush are presented in Table 3. The FRAP value of the oven-dried spiny saltbush (81.92 ± 3.32 μmol TEAC eq/g DW) was significantly higher (*p* < 0.05) than that of the freeze-dried spiny saltbush (59.12 ± 0.49 μmol TEAC eq/g DW). This phenomenon might be due to the release or activation of antioxidant phenolic constituents during the heating process. FRAP was increased by oven drying, causing polyphenol oxidases to break down more rapidly. Moreover, the release of polyphenols bound to other compounds (proteins, oligo- or polysaccharides a) or chemically modified to release phenols, thereby increasing their antioxidant properties. During freeze-drying, lower temperatures maintain certain sensitive compounds’ stability, but prolonged exposure to freezing conditions or crystallization pressure may result in antioxidant loss or hinder their release [41,43].

**Figure 5:**
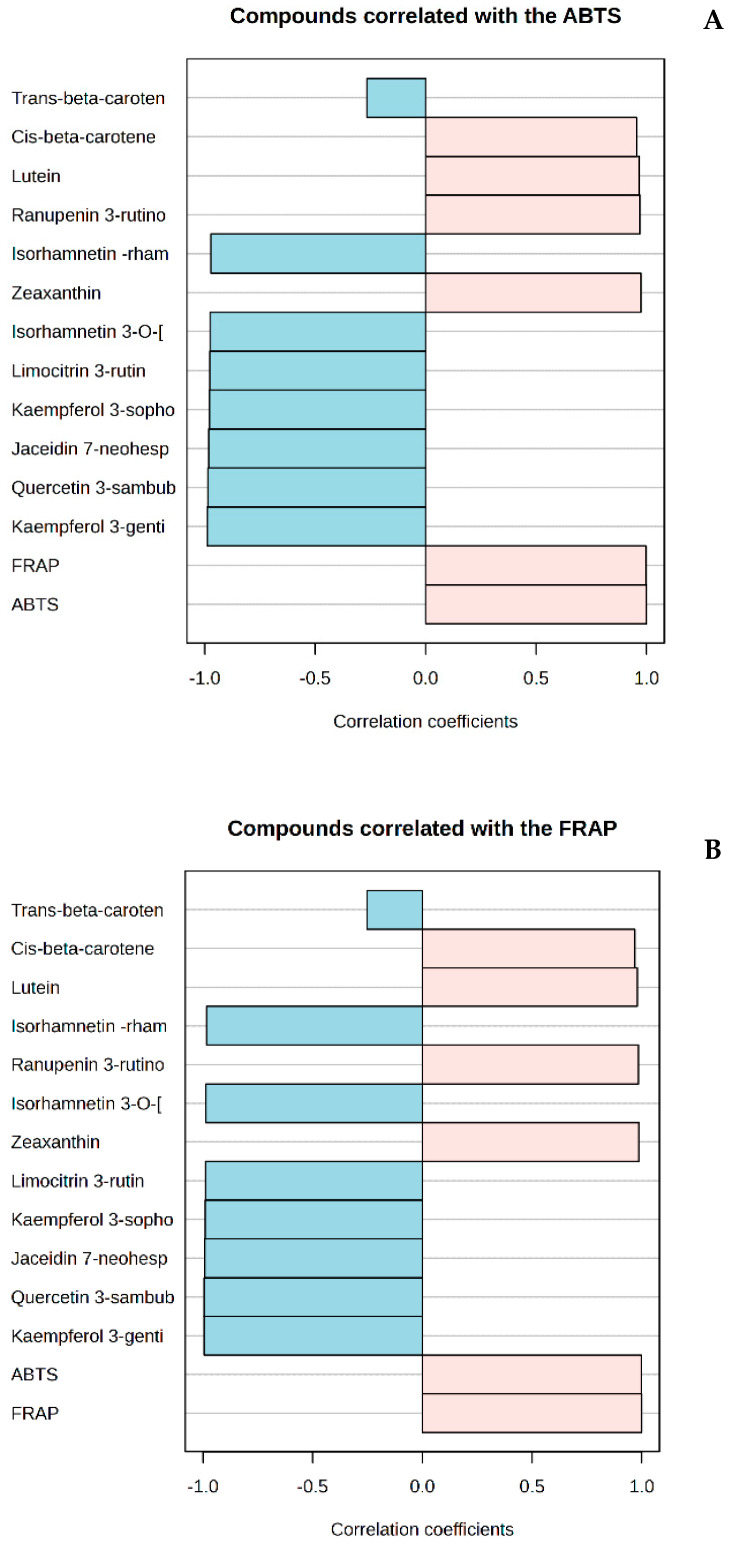
Correlation between ABTS (**A**), FRAP (**B**) and bioactive compounds (phenolics and carotenoids) in freeze-dried and oven-dried spiny saltbush.

Furthermore, the drying method significantly influenced the ABTS scavenging activity, with the ABTS activity of the oven-dried material (75.08 ± 12.83 μmole TEAC/g DW) being significantly (*p* < 0.05) higher than that of the freeze-dried material (28.44 ± 1.09 μmole TEAC/g DW). It is possible that oven-dried samples have higher ABTS and FRAP antioxidant activity because their higher temperatures allow compounds with antioxidant effects, such as phenolic substances, to be released more effectively. Meanwhile, long-term low temperatures during freeze-drying can partially degrade antioxidants [43]. Oven-dried samples with higher carotenoid content may have greater ABTS scavenging abilities due to molecular changes such as converting cyclic carotenoids to acyclic carotenoids [44]. Figure 5A,B and Tables S6 and S7 show a significant correlation between ABTS or FRAP and metabolites (bioactive compounds). The strongest positive correlation of FRAP with bioactive compounds (*p* < 0.05) was with zeaxanthin (*r* = 0.98628), followed by ranupenin 3-rutinoside (*r* = 0.98375), lutein (*r* = 0.97983), cis-β carotene (*r* = 0.96884), and quercetin 3-galactoside (*r* = 0.44396). The rest of the compounds showed a negative correlation. A similar trend was observed for the correlation between ABTS and the same compounds.

### 3.7. Effect of Drying Method on In Vitro Cell Viability

**Figure 6:**
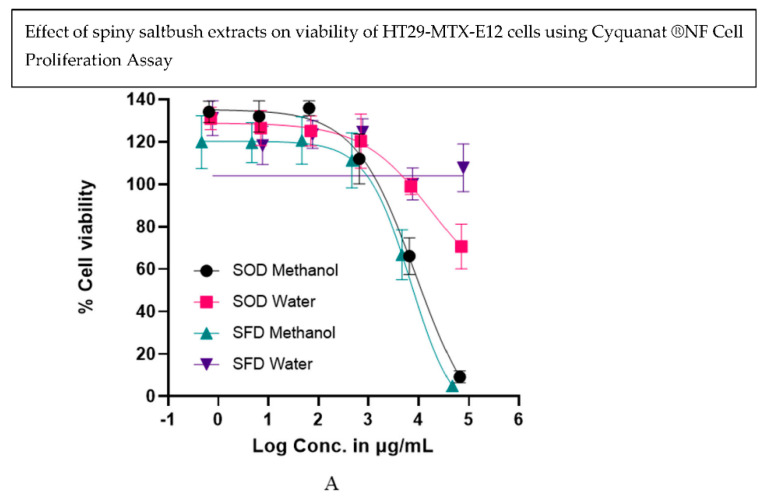

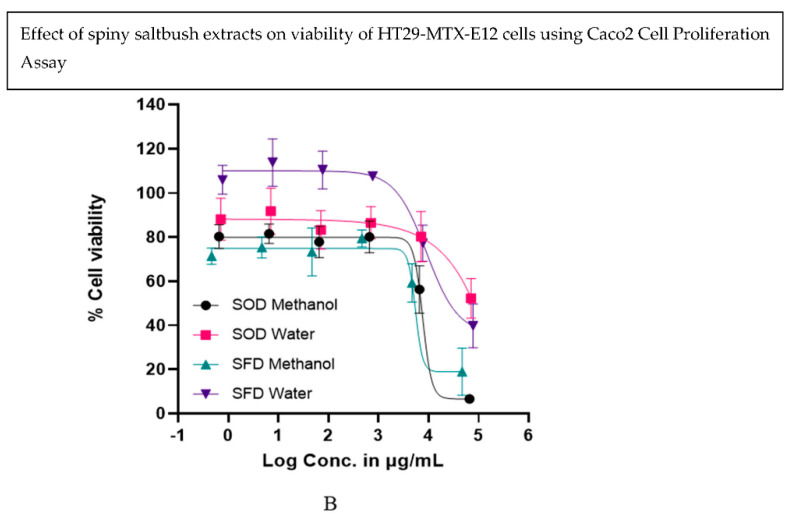
Treatment concentration–response curves for spiny saltbush oven-dried (SOD) and spiny saltbush freeze-dried (SFD) methanol and water extracts expressed as percentage viable intestinal cells, HT29-MTX-E12 (**A**) and Caco2 (**B**) remaining after 2 h treatment compared to the HBSS control in CyQUANT^®^ NF Cell Proliferation Assay. The data are presented as the mean percentage (±SEM) of three replicates for each treatment.

The treatment-concentration response curves of the oven and freeze-dried spiny saltbush sample extracts are presented in Figure 6A,B. Among the two different extracts, the methanolic extract demonstrated higher cytotoxicity against both Caco-2 and HT-29 cell lines, indicating that methanol may be more effective in extracting bioactive compounds. The methanol extract of freeze-dried samples (SFD) showed the lowest IC_50_ values (7010 μg/mL and 5440 μg/mL) against HT-29 and Caco-2 cell lines, respectively, while the methanol extract of oven-dried spiny saltbush (SOD) displayed IC_50_ values of 8756 μg/mL and 7606 µg/mL against HT-29 and Caco-2 cell lines, respectively. Despite applying very high concentrations of the water extracts, it was not possible to obtain an IC_50_ value for the SFD water extracts in HT29 cells as the cell viability remained almost 100% with the highest concentration applied (76,557 µg/mL) and more than 50% cell viability was achieved with the highest concentration of the SOD water extracts (70,563 µg/mL) in Caco2 cells. This could be due to the non-toxic nature of the bioactive compounds released in the water extracts. A previous study on the extracts of *Atriplex confertifolia* (a plant native to North America, commonly referred to as shadscale or saltbush) has also reported the non-toxic nature of the plant extracts on the viability of human breast cancer cell lines MCF-7, MDA-MB 435, MDA-MB 231, and HeLa cells [45]. The IC_50_ values obtained on the SOD and SFD methanol extracts against Caco2 and HT29 cells are also comparable to the other Australian native fruits [45], indicating minimal impact and negligible toxicity on the spiny saltbush extracts. Spiny saltbush oven-dried samples had higher antioxidant activity than freeze-dried samples and did not influence the percent viability of HT29 and Caco-2 cells. However, according to the heatmap of both drying methods (Figure 4B), the freeze-dried sample accumulated more bioactive compounds than the oven-dried, which may explain its higher activity on the viability of the cells as compared to the oven-dried samples. However, these extracts cannot be considered as having cytotoxic activity because their IC_50_ values were >500 µg/mL [45]. This is the first report to show that spiny saltbush extracts are safe to be used on mammalian intestinal cells in vitro. Cell viability was not influenced by water extracts of oven-dried or freeze-dried samples, and only very high concentrations of methanol extracts inhibited cell viability.

## 4. Conclusions

This study examined the changes in phytonutrient compounds and antioxidant activity in spiny saltbush (*Rhagodia spinescens*) during oven and freeze-drying. During freeze-drying, phenolic metabolites were more abundant, while carotenoid components (lutein and beta carotene) were less abundant. OPLS-DA and PCA results indicated that drying methods separated samples into two distinct groups based on VIP > 1. Among the differential metabolites, dehydrophytosphingosine (7.971/316.2848), kaempferol 3-sophorotrioside (3.948/773.2156), and quercetin 3-sambubioside-7-glucoside (4.06/759.200) separated freeze-dried and oven-dried spiny saltbush. Compared to oven-dried spiny saltbush, freeze-dried samples contained more phenolic glycosides. The oven-dried samples contained high levels of lutein, trans and cis β-carotene, and exhibited significant in vitro antioxidant activity. Finally, phenolic and carotenoid metabolites were correlated with antioxidant activities. Additionally, both drying methods produced non-toxic samples. According to the findings, hot air oven drying is a more cost-effective method than freeze drying for improving nutritional compounds, carotenoid components, and antioxidant activity. Also provides insight into the changes in phytonutrients, metabolites, and antioxidant activity to select the most cost-effective method to produce high-quality dried spiny saltbush. The results show that spiny saltbush can be used as a salt alternative or functional ingredient in the food industry.

## Figures and Tables

**Table 1:** Minerals, trace elements, and proximate content of oven-dried and freeze-dried spiny saltbush.

Parameters	Oven-Dried Spiny Saltbush	Freeze-Dried Spiny Saltbush
Na (g/100 g dw)	4.72 ± 0.45 ^a^	3.86 ± 0.31 ^b^
K (g/100 g dw)	6.86 ± 0.34 ^a^	4.46 ± 0.23 ^b^
Ca (g/100 g dw)	4.06 ± 0.12 ^a^	2.83 ± 0.41 ^b^
Mg (g/100 g dw)	1.19 ± 0.22 ^ns^	1.15 ± 0.23 ^ns^
Fe (mg/kg dw)	149 ± 0.30 ^ns^	149 ± 0.16 ^ns^
Zn (mg/kg dw)	372 ± 0.21^a^	299 ± 0.19 ^a^
Total fiber (mg/100 g)	54,110 ± 0.74 ^b^	46,240 ± 2.07 ^a^
Moisture (%)	3.44 ± 0.84 ^b^	10.56 ± 0.25 ^a^
Water activity	0.24 ± 0.01 ^b^	0.58 ± 0.01 ^a^
Colour (ΔE*)	12.63 ± 1.78 ^ns^	14.93 ± 3.24 ^ns^
Protein (mg/100 g)	11,810 ± 0.20 ^a^	8000 ± 0.40 ^b^
Fat (%)	25.90 ± 0.56 ^b^	39.70 ± 0.02 ^a^
Ash (%)	28,790 ± 0.06 ^b^	33,350 ± 0.22 ^a^

Results are expressed as mean ± SD of triplicate measurements. ns—significant. The values in the same row followed by different letters are significantly different at *p* < 0.05. * Colour change.

**Table 2:** Phenolic compounds identified in spiny saltbush (*(Rhagodia spinescens)* by UPLC-MS/MS.

N°	RT	[M-H]^−^ (*m*/*z*)	MSE Fragments	Molecular Formula	Identified Compound	Concentrations in mg/kg in the Solid Versus Rutin Calibration Curve	
						Oven-Dried Spiny Saltbush	Freeze-Dried Spiny Saltbush
1	3.948	773.2156	333.0604 465.1031 627.1555 765.1896 773.2153	C_33_H_40_O_21_	Kaempferol 3-sophorotrioside	9.965 ± 0.436 ^b^	350.135 ± 9.040 ^a^
2	4.06	759.2001	303.0492 317.0660 333.0608 449.1084 465.1033 627.1562 759.2000	C_32_H_38_O_21_	Quercetin 3-sambubioside-7-glucoside	9.686 ± 1.432 ^b^	273.551 ± 2.038 ^a^
3	4.066	757.2213	317.0662 333.0618 449.1100 611.1622 757.2203	C_33_H_40_O_20_	Kaempferol 3-gentiobioside 7-rhamnoside	59.232 ± 1.852 ^b^	115.373 ± 0.990 ^a^
4	4.153	787.2315	332.0535 347.0766 479.1190 641.1710 787.2305	C_34_H_42_O_21_	Isorhamnetin-rhamnosyl-hexoside-glucoside	766.106 ± 12.747 ^b^	1499.369 ± 81.628 ^a^
5	4.171	787.2318	332.0535 347.0766 479.1190 641.1710 787.2305	C_34_H_42_O_21_	Isorhamnetin 3-O-[b-D-glucopyranosyl-(1->2)-[a-L-rhamnopyranosyl-(1->6)]-b-D-glucopyranoside]	745.276 ± 14.105 ^b^	1438.388 ± 65.549 ^a^
6	4.537	641.1721	303.0501 347.0761 479.1190 641.1727	C_28_H_32_O_17_	Ranupenin 3-rutinoside	97.966 ± 3.190 ^a^	35.035 ± 0.711 ^b^
7	4.817	655.1877	332.0528 347.0762 509.1286 575.1719 655.1863	C_29_H_34_O_17_	Limocitrin 3-rutinoside	15.682 ± 0.251 ^b^	123.611 ± 3.637 ^a^
8	5.495	669.2037	346.0689 361.0922 523.1462 669.2022	C_30_H_36_O_17_	Jaceidin 7-neohesperidoside	182.444 ± 3.864 ^b^	458.185 ± 10.734 ^a^

Values represent the mean ± standard deviation. The values in the same row followed by different letters are significantly different at *p* < 0.05.

**Table 3:** Effect of drying methods on carotenoids and the antioxidant activity of spiny saltbush.

Carotenoid Components	Freeze-Dried Spiny Saltbush	Oven-Dried Spiny Saltbush
Total carotenoids (mg/100 g)	3804.33 ± 278.09 ^a^	2731.14 ± 403.18 ^b^
Lutein (mg/100 g)	49.24 ± 0.88 ^b^	64.48 ± 0.91 ^a^
Trans-β carotene (mg/100 g)	2248.41 ± 80.03 ^a^	2218.91 ± 88.66 ^a^
Cis-β-carotene (mg/100 g)	151.75 ± 10.88 ^b^	299.54 ± 21.27 ^a^
Zeaxanthin (mg/100 g)	0 ^b^	1.67 ± 0.07 ^a^
**Antioxidant Activity**		
ABTS (μmol TEAC eq/g DW)	28.44 ± 1.09 ^b^	75.08 ± 12.83 ^a^
FRAP (μmol TEAC eq/g DW)	59.12 ± 0.49 ^b^	81.92 ± 3.32 ^a^

Values represent the mean ± standard deviation. The values in the same row followed by different letters are significantly different at *p* < 0.05.

## Data Availability

Data can be made available after approval from the Postgraduate studies unit of university of Queensland.

## Supplementary Materials

The following supporting information can be downloaded at https://www.mdpi.com/article/10.3390/antiox13111382/s1, Table S1: Moisture content, chlorophyll content, and color of fresh spiny saltbush leaves. Table S2: UPLC-Q-TOF-MS/MS metabolites’ identification, Table S3: Metabolites’ loadings on PCA plot score; Table S4: Metabolites’ loading on PLS-DA plot score; Table S5: Metabolites’ VIP score; Table S6: Phenolic and carotenoids compounds correlations with FRAP; Table S7: Phenolic and carotenoids compounds correlation with ABTS; Figure S1: UPLC-QTOF/MS BPI chromatogram in ESI negative mode of oven-dried spiny saltbush; Figure S2: UPLC-QTOF/MS BPI chromatogram in ESI negative mode of freeze-dried spiny saltbush; Figure S3: MS/MS spectrum of Kaempferol 3-sophorotrioside adjacent to its chemical structure; Figure S4: MS/MS spectrum of Quercetin 3-sambubioside-7-glucoside adjacent to its chemical structure; Figure S5: MS/MS spectrum of Kaempferol 3-gentiobioside 7-rhamnoside adjacent to its chemical structure; Figure S6: MS/MS spectrum of Isorhamnetin 3-O-[b-D-glucopyranosyl-(1->2)-[a-L-rhamnopyranosyl-(1->6)]-b-D-glucopyranoside] adjacent to its chemical structure; Figure S7: MS/MS spectrum of Ranupenin 3-rutinoside and its chemical structure; Figure S8: MS/MS spectrum of Limocitrin 3-rutinoside with its chemical structure; Figure S9: MS/MS spectrum of Jaceidin 7-neohesperidoside with its chemical structure.
