# Supplementary material for: Impact of Drying on Phytonutritional Compounds, In Vitro Antioxidant Activity and Cytotoxicity of Spiny Saltbush (Rhagodia spinescens)
Source: Antioxidants (Basel). 2024 Nov 12;13(11):1382. doi: 10.3390/antiox13111382 (PMC11591164; doi:10.3390/antiox13111382)
Supplement: Supplementary file 1 [file antioxidants-13-01382-s001.zip › antioxidants-3295558-supplementary.pdf]

Supplementary Table S1: Moisture content, chlorophyll content, and color of fresh spiny saltbush leaves.

**Parameters**	**Spiny saltbush fresh leaves**
Moisture content (%)	87.19 ± 0.395
Chlorophyl a (mg/g FW)	0.05 ± 0.004
Chlorophyll b (mg/g FW)	0.02 ± 0.002
Total Chlorophyll (mg/g FW)	0.07 ± 0.006
Colour:	
*L**	47.86
*a**	-8.87
*b**	13.17

Data are means ± SD (n = 3). FW: fresh weight.

Supplementary Table S2: UPLC-Q-TOF-MS/MS metabolites' identification

**Alignment ID**	**Average Rt(min)**	**Average Mz**	**Structure rank 1**	**Total score**	**Databases**	**Formula**	**Ontology**	**MS/MS spectrum**
350	2.773	613.1606	MINEs-158560	2.569	MINE=Cba6faf34d6163f1d524d35588fe59944923a556f	C12H18O7S	Thioglycosides	207.12405:118 224.12917:676 231.04568:207 235.11954:9445 236.12219:1290 268.10526:1160 314.12491:435 330.1124:822 332.13376:2049 333.12759:105 333.14053:105 355.07391:465 459.15302:1375 459.20215:1 460.16046:136 484.11813:282 613.10986:15 613.15857:1289
40	3.144	249.124	Abbeymycin	6.4772	HMDB=HMDB0004081, ChEBI=CHEBI:2198, SMPDB=PW_C002052, FooDB=FDB023304, BMDB=BMDB04081, Urine=HMDB0004081, Serum=HMDB0004081, PubChem=1864, PlantCyc=CPD-12014,	C13H16N2O3	Hydroxyindoles	203.1187:950 249.12483:1645

					BLEXP=BLEXPDB00000001864, COCONUT=CNP0349869			
78	3.494	298.0974	5'-Methylthioadenosine	6.8004	HMDB=HMDB0001173, ChEBI=CHEBI:17509, DrugBank=DB02282, SMPDB=PW_C000910, YMDB=YMDB00178, FooDB=FDB022465; FDB031156, BMDB=BMDB01173, Urine=HMDB0001173, ECMDB=ECMDB01173, Serum=HMDB0001173, PubChem=439176, PlantCyc=5-METHYLTHIOADENOSINE, UNPD=UNPD190794; UNPD27064, BLEXP=BLEXPDB00000439176, NPA=NPA008075, COCONUT=CNP0141020	$C_{11}H_{15}N_5O_3S$	5'-deoxy-5'-thionucleosides	188.07121:971<br>298.09714:2616
15	3.496	1066.75	Peptide					188.07121:971
398	3.948	773.2156	Kaempferol 3-sophorotrioside	6.6972	HMDB=HMDB0032008, KNApSAcK=C00005208; C00013750, ChEBI=CHEBI:31744, FooDB=FDB008706; FDB016669, LipidMAPS=LMPK12111744; LMPK12111918, PubChem=90658031; 5282156, PlantCyc=CPD-8068, UNPD=UNPD124216;	$C_{33}H_{40}O_{21}$	Flavonoid-3-O-glycosides	318.03732:843<br>333.06049:17984<br>334.06387:2989<br>335.04831:530<br>335.06732:525<br>347.07632:208<br>435.09229:43<br>436.05527:11<br>436.14154:1<br>465.10318:8091<br>466.10715:1742

					UNPD17579; UNPD72804, COCONUT=CNP0174298			467.10699:426<br>597.20911:5<br>598.20032:2<br>627.07312:2<br>627.15558:5436<br>628.14191:1681<br>628.16168:1705<br>628.22662:4<br>629.15942:412<br>629.21167:1<br>663.11243:5<br>663.14862:76<br>663.17151:76<br>680.01904:7<br>680.06921:209<br>681.07678:95<br>744.14075:15<br>765.18964:132<br>765.2569:3<br>773.11139:2<br>773.21539:3780
395	4.06	759.200<br>1	Quercetin 3-sambubioside-7-glucoside	5.648<br>4	KNApSAcK=C00005462, LipidMAPS=LMPK12112116, UNPD=UNPD516, COCONUT=CNP0107701	C32H38O21	Flavonoid-7-O-glycosides	302.04251:87<br>303.04922:1733<br>304.05475:149<br>317.02759:56<br>317.06601:6265<br>318.03781:520<br>318.06973:1019<br>333.06085:14292<br>334.06421:2378<br>335.05508:290<br>335.06952:290<br>411.16525:787

								435.09354:1265<br>436.07422:172<br>436.0997:222<br>449.10849:3208<br>450.11267:730<br>465.04678:3<br>465.10333:6370<br>466.10529:1348<br>467.10815:232<br>467.12482:225<br>597.12:205<br>597.12842:296<br>597.14844:294<br>611.16071:1404<br>611.22076:9<br>612.16614:412<br>627.15625:3199<br>628.15717:903<br>628.21637:2<br>629.16376:64<br>729.15118:15<br>729.19269:199<br>751.15387:155<br>751.17719:160<br>757.133:5<br>757.19025:72<br>757.22351:1068<br>758.15479:9<br>758.20148:348<br>758.22852:418<br>759.10767:6<br>759.20007:3159

394	4.066	757.2213	Kaempferol 3-gentiobioside 7-rhamnoside	6.9324	HMDB=HMDB0034396, KNApSAcK=C00005234, ChEBI=CHEBI:133218, FooDB=FDB006679; FDB012785, LipidMAPS=LMPK12111762, PubChem=25203808, PlantCyc=CPD1F-431, UNPD=UNPD85608; UNPD86882; UNPD94668, COCONUT=CNP0322412	C33H40O20	Flavonoid-7-O-glycosides	317.06622:3881 318.07043:618 333.06186:700 411.16122:467 411.18988:7 449.11008:1906 450.11627:333 465.10339:134 611.10913:6 611.16229:866 612.16656:159 757.14801:8 757.22034:600
400	4.171	787.2318	Isorhamnetin 3-O-[b-D-glucopyranosyl-(1->2)-[a-L-rhamnopyranosyl-(1->6)]-b-D-glucopyranoside]	6.6693	HMDB=HMDB0038254, KNApSAcK=C00005567; C00005572,FooDB=FDB017547, LipidMAPS=LMPK12112327; LMPK12112349, UNPD=UNPD161985; UNPD163679; UNPD186202; UNPD214560, COCONUT=CNP0292525	C34H42O21	Flavonoid-3-O-glycosides	317.06543:17 332.05356:1512 347.04541:30 347.07669:41090 348.08032:7099 349.08307:1104 377.14526:285 479.11902:19931 479.16687:2 480.12344:4401 481.12445:911 509.1293:202 562.01215:33 562.04272:170 563.04871:310 641.08154:9 641.17108:13155 642.17456:3795 643.1239:34 643.17401:928

								643.19586:927<br>643.24359:3<br>677.10876:7<br>677.13409:41<br>677.17133:341<br>677.18787:341<br>677.22467:3<br>694.03979:31<br>694.07349:336<br>694.0965:388<br>694.13464:7<br>695.02911:29<br>695.05176:29<br>695.09143:334<br>695.13159:4<br>787.1214:2<br>787.18097:152<br>787.23053:11190<br>787.31604:5
24	4.393	209.117<br>9	Elemicin	7.272<br>6	HMDB=HMDB0033778,<br>KNApSAcK=C00002739,<br>ChEBI=CHEBI:4771,<br>FooDB=FDB011932,<br>NANPDB=NANPDB_4344;<br>NANPDB_4359,<br>UNPD=UNPD81543,<br>BLEXP=BLEXPDB00000010248<br>, COCONUT=CNP0286854	C12H16O3	Anisoles	177.05515:2641<br>209.11821:735
364	4.537	641.172<br>1	Ranupenin 3-<br>rutinoside	7.216<br>7	HMDB=HMDB0037358,<br>KNApSAcK=C00005701,<br>FooDB=FDB016385,<br>LipidMAPS=LMPK12113184,	C28H32O17	Flavonoid-3-O-<br>glycosides	303.05014:805<br>332.05423:245<br>347.07614:6420<br>348.0784:1128<br>479.11902:1562

					UNPD=UNPD145128, COCONUT=CNP0228182			479.16342:11 480.12106:277 641.17273:1420
372	4.817	655.187 7	Limocitrin 3-rutinoside	5.862 4	HMDB=HMDB0037579, KNApSAcK=C00005719, FooDB=FDB016680, LipidMAPS=LMPK12113191, UNPD=UNPD119318; UNPD172479; UNPD22939, COCONUT=CNP0283196	C29H34O17	Flavonoid-3-O-glycosides	332.0528:1571 333.05917:412 347.07629:15576 347.11807:5 348.08057:2623 349.07932:348 349.09454:335 503.27826:21 503.30594:154 509.12869:1148 520.33258:273 575.17194:1188 576.17786:354 605.1842:1205 606.16431:125 606.17596:373 606.19562:311 655.12866:11 655.18634:551
276	5.013	481.316 7	Crustecdysone	7.053 3	HMDB=HMDB0030180, KNApSAcK=C00003654; C00033543;C00048286; C00048292;C00049773, ChEBI=CHEBI:16587, FooDB=FDB001996, NANPDB=NANPDB_1303; NANPDB_2918; NANPDB_3353, LipidMAPS=LMST01010209, PubChem=439770;21362,	C27H44O7	Hydroxy bile acids, alcohols and derivatives	301.17902:292 371.22195:1490 372.22348:264 409.27481:505 427.24292:4 427.28497:1828 428.28876:479 445.29523:3397 446.29871:941 463.30673:894

					UNPD=UNPD107802; UNPD113001; UNPD126415; UNPD135839; UNPD147324; UNPD185067; UNPD189991; UNPD28189; UNPD30699; UNPD37447; UNPD52113; UNPD7032; UNPD78702; UNPD79493; UNPD86729, BLEXP=BLEXPDB00000271605 , COCONUT=CNP0146238			464.31342:62 481.31537:1235
380	5.495	669.203 7	Jaceidin 7-neohesperidoside	6.043 1	KNApSAcK=C00005674, LipidMAPS=LMPK12112932, UNPD=UNPD50426, COCONUT=CNP0282232	C30H36O17	Flavonoid-7-O-glycosides	346.06894:495 361.04288:3 361.09222:18387 361.13748:2 362.09601:3193 363.07004:13 363.09515:365 523.14624:2318 523.19586:8 524.10126:21 524.12543:487 524.14929:570 524.18231:25 669.1355:15 669.20227:964 669.26282:3
421	5.603	972.515 8	Unknown					339.1412:1559 611.35938:316 972.51221:2122 972.61725:7 972.71985:1 972.7699:48

								972.84625:3627<br>972.94666:6
362	5.955	638.209<br>5	Unknown					177.05513:3434<br>178.12386:256<br>207.06581:8<br>353.08798:3865<br>354.09149:645<br>354.12384:4<br>361.08963:233<br>427.12567:1581<br>449.10538:429<br>545.33124:273<br>621.1853:410<br>621.23529:3<br>638.19073:309<br>638.21472:320
433	6.039	1438.24<br>5	Peptide					177.05511:760<br>353.06427:414<br>353.0889:463<br>669.40228:38<br>700.41254:721<br>813.4566:27<br>813.48425:130<br>813.51123:140<br>958.73016:11<br>958.75159:9<br>958.82043:521<br>958.90125:3<br>959.08484:21<br>959.09534:30<br>959.15393:836<br>959.41138:13<br>959.49292:764

								959.5871:1<br>959.8208:367<br>959.84149:367<br>959.87079:10<br>960.10114:5<br>960.1557:93<br>1437.54309:3<br>1437.7334:2515<br>1437.86548:12<br>1437.90076:8<br>1438.04651:6<br>1438.23376:4186<br>1438.39465:10<br>1438.54041:7<br>1438.73804:3647
102	7.971	Dehydrophytosphingosine		7.351<br>1	HMDB=HMDB0038057,<br>ChEBI=CHEBI:20386;<br>CHEBI:83175,<br>FooDB=FDB017268,<br>LipidMAPS=LMSP01030002;<br>LMSP01080009,<br>PlantCyc=CPD-10693,<br>UNPD=UNPD107172;<br>UNPD142582; UNPD67326,<br>BLEXP=BLEXPDB00014757418<br>, COCONUT=CNP0104894	C18H37NO3	1,3-<br>aminoalcohols	QSBHSPFCUOHNK<br>Q-ZLKVKTCBNA-N
303	9.897	520.341	Sespendole	4.291<br>3	NPA=NPA009868,<br>COCONUT=CNP0305219	C33H45NO4	3-alkylindoles	296.29498:1869<br>326.38004:437<br>359.23981:465<br>421.3403:446<br>520.29413:18<br>520.33911:1708<br>520.39264:6

Supplementary Table S3: Metabolites' loadings on PCA plot score

Peak	Compound name	PC1	PC2
2.773/613.1605	MINEs-158560	-0.25867	0.057352
3.144/249.1239	Abbeymycin	0.078338	0.21707
3.494/298.0973	5'-Methylthioadenosine	-0.26441	0.29713
3.496/1066.75	Peptide	-0.11787	0.11686
3.948/773.2156	Kaempferol 3-sophorotrioside	-0.38044	0.10153
4.06/759.2001	Quercetin 3-sambubioside-7-glucoside	-0.36872	0.12686
4.066/757.2212	Kaempferol 3-gentiobioside 7-rhamnoside	-0.16457	0.065488
4.153/787.2313	Isorhamnetin -rhamnosyl-hexoside-glucoside	-0.1643	0.18969
4.171/787.2318	Isorhamnetin 3-O-[b-D-glucopyranosyl-(1->2)-[a-L-rhamnopyranosyl-(1->6)]-b-D-glucopyranoside]	-0.16287	0.15395
4.393/209.1179	Elemicin	-0.12285	0.014209
4.537/641.1721	Ranupenin 3-rutinoside	0.20437	-0.02349
4.817/655.1876	Limocitrin 3-rutinoside	-0.28966	0.096583
5.013/481.3166	Crustecdysone	0.053726	-0.2127
5.495/669.2036	Jaceidin 7-neohesperidoside	-0.19342	0.079866
5.603/972.5158	Unknown	-0.13483	-0.15041
5.955/638.2094	Unknown	-0.31511	0.1244
6.039/1438.245	Peptide	-0.09724	0.11878
7.971/316.2848	Dehydrophytosphingosine	0.41697	0.8021

Supplementary Table S4: Metabolites' loading on PLS-DA plot score

Peak	Compound name	Comp 1	Comp 2
2.773/613.1605	MINEs-158560	-0.25867	-0.06097
3.144/249.1239	Abbeymycin	0.078332	-0.21874
3.494/298.0973	5'-Methylthioadenosine	-0.26442	-0.29649
3.496/1066.75	Peptide	-0.11788	-0.11441
3.948/773.2156	Kaempferol 3-sophorotrioside	-0.38045	-0.10142
4.06/759.2001	Quercetin 3-sambubioside-7-glucoside	-0.36873	-0.12411
4.066/757.2212	Kaempferol 3-gentiobioside 7-rhamnoside	-0.16457	-0.06218
4.153/787.2313	Isorhamnetin -rhamnosyl-hexoside-glucoside	-0.16431	-0.19209
4.171/787.2318	Isorhamnetin 3-O-[b-D-glucopyranosyl-(1->2)-[a-L-rhamnopyranosyl-(1->6)]-b-D-glucopyranoside]	-0.16287	-0.15334
4.393/209.1179	Elemicin	-0.12285	-0.01341
4.537/641.1721	Ranupenin 3-rutinoside	0.20437	0.027947
4.817/655.1876	Limocitrin 3-rutinoside	-0.28966	-0.09733
5.013/481.3166	Crustecdysone	0.053733	0.22804

5.495/669.2036	Jaceidin 7-neohesperidoside	-0.19342	-0.07724
5.603/972.5158	Unknown	-0.13483	0.15555
5.955/638.2094	Unknown	-0.31511	-0.12776
6.039/1438.245	Peptide	-0.09724	-0.10747
7.971/316.2848	Dehydrophytosphingosine	0.41695	-0.80831

Supplementary Table S5: Metabolites' VIP score

Peak	Compound name	Comp. 1	Comp. 2
7.971/316.2848	Dehydrophytosphingosine	1.7593	1.7612
3.948/773.2156	Kaempferol 3-sophorotrioside	1.6153	1.6147
4.06/759.2001	Quercetin 3-sambubioside-7-glucoside	1.5657	1.5651
5.955/638.2094	Unknown	1.3387	1.3383
4.817/655.1876	Limocitrin 3-rutinoside	1.2301	1.2297
3.494/298.0973	5'-Methylthioadenosine	1.1252	1.1252
2.773/613.1605	MINEs-158560	1.0985	1.0981
4.537/641.1721	Ranupenin 3-rutinoside	0.86779	0.86745
5.495/669.2036	Jaceidin 7-neohesperidoside	0.82134	0.82102
4.153/787.2313	Isorhamnetin -rhamnosyl-hexoside-glucoside	0.6995	0.69961
4.066/757.2212	Kaempferol 3-gentiobioside 7-rhamnoside	0.69876	0.69848
4.171/787.2318	Isorhamnetin 3-O-[b-D-glucopyranosyl-(1->2)-[a-L-rhamnopyranosyl-(1->6)]-b-D-glucopyranoside]	0.69274	0.69267
5.603/972.5158	Unknown	0.57007	0.57016
4.393/209.1179	Elemicin	0.52132	0.52111
3.496/1066.75	Peptide	0.50116	0.50106
6.039/1438.245	Peptide	0.41309	0.41295
3.144/249.1239	Abbeymycin	0.3296	0.33058
5.013/481.3166	Crustecdysone	0.23183	0.23487

Supplementary Table S6: Phenolic and carotenoids compounds correlations with FRAP

Compounds	correlation	t-stat	p-value	FDR
FRAP	1	94906000	7.40E-32	1.04E-30
ABTS	0.99794	31.142	6.34E-06	4.44E-05
Kaempferol 3-gentiobioside 7-rhamnoside	-0.99534	-20.64	3.26E-05	0.000152
Quercetin 3-sambubioside-7-glucoside	-0.99426	-18.584	4.93E-05	0.000173
Jaceidin 7-neohesperidoside	-0.99209	-15.804	9.37E-05	0.000262
Kaempferol 3-sophorotrioside	-0.98946	-13.668	0.000166	0.000386
Limocitrin 3-rutinoside	-0.98863	-13.152	0.000193	0.000386

Zeaxanthin	0.98628	11.951	0.000281	0.000462
Isorhamnetin 3-O-[b-D-glucopyranosyl-(1->2)-[a-L-rhamnopyranosyl-(1->6)]-b-D-glucopyranoside]	-0.9859	-11.782	0.000297	0.000462
Ranupenin 3-rutinoside	0.98375	10.96	0.000394	0.00055
Isorhamnetin -rhamnosyl-hexoside-glucoside	-0.98298	-10.7	0.000432	0.00055
Lutein	0.97983	9.8067	0.000606	0.000707
Cis-beta-carotene	0.96884	7.8231	0.001441	0.001552
Trans-beta-carotene	-0.25281	-0.5226	0.62886	0.62886

Supplementary Table S7: Phenolic and carotenoids compounds correlation with ABTS

**Compounds**	**correlation**	**t-stat**	**p-value**	**FDR**
ABTS	1	Inf	0	0
FRAP	0.99794	31.142	6.34E-06	4.44E-05
Kaempferol 3-gentiobioside 7-rhamnoside	-0.98731	-12.435	0.00024	0.001105
Quercetin 3-sambubioside-7-glucoside	-0.98546	-11.6	0.000316	0.001105
Jaceidin 7-neohesperidoside	-0.98228	-10.482	0.000468	0.001311
Kaempferol 3-sophorotrioside	-0.97837	-9.4595	0.000697	0.001558
Limocitrin 3-rutinoside	-0.97703	-9.17	0.000785	0.001558
Isorhamnetin 3-O-[b-D-glucopyranosyl-(1->2)-[a-L-rhamnopyranosyl-(1->6)]-b-D-glucopyranoside]	-0.97505	-8.7845	0.000926	0.001558
Zeaxanthin	0.97405	8.6073	0.001001	0.001558
Isorhamnetin -rhamnosyl-hexoside-glucoside	-0.97134	-8.1735	0.00122	0.001631
Ranupenin 3-rutinoside	0.97063	8.0691	0.001281	0.001631
Lutein	0.96689	7.5775	0.001627	0.001898
Cis-beta-carotene	0.95568	6.4921	0.002903	0.003126
Trans-beta-carotene	-0.26444	-0.5484	0.61259	0.61259

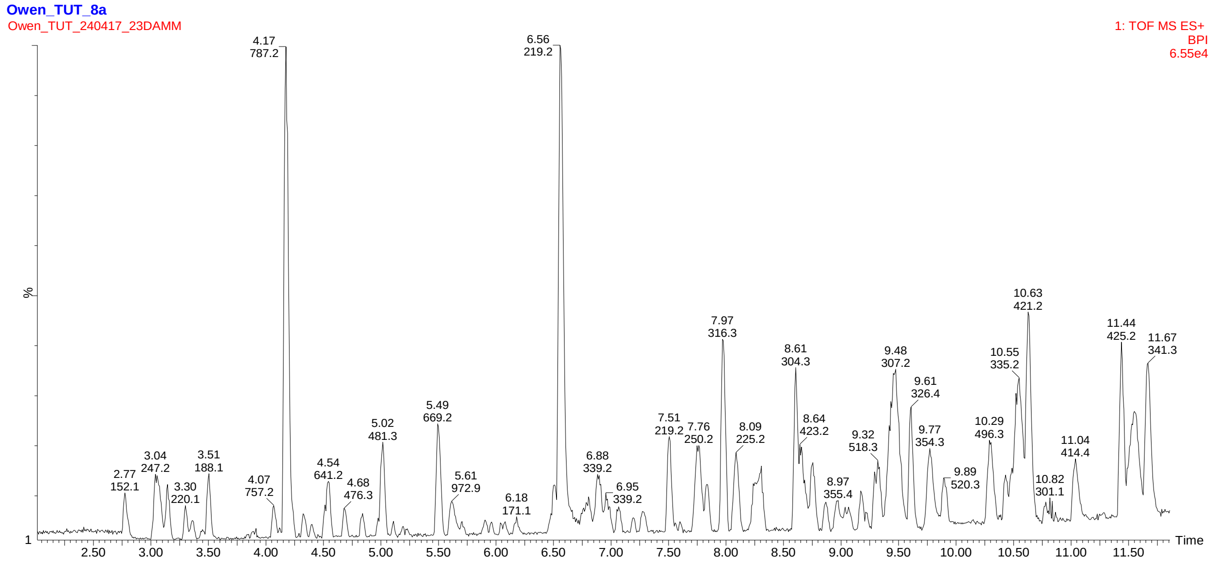

Supplementary Figure S1: UPLC-QTOF/MS BPI chromatogram in ESI negative mode of oven-dried spiny saltbush

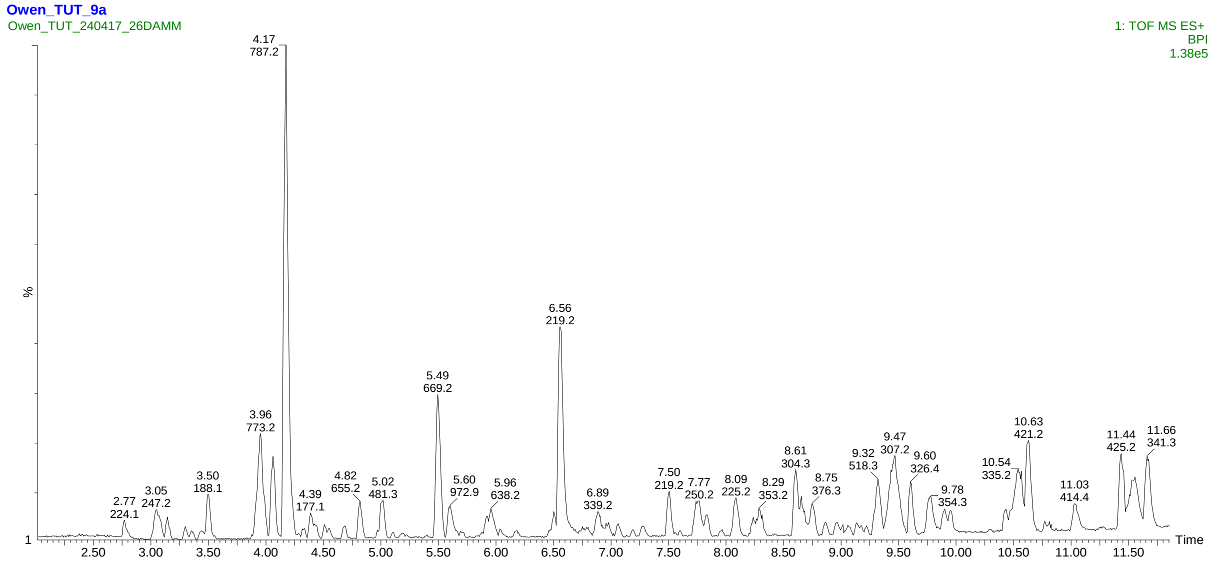

Supplementary Figure S2: UPLC-QTOF/MS BPI chromatogram in ESI negative mode of freeze-dried spiny saltbush

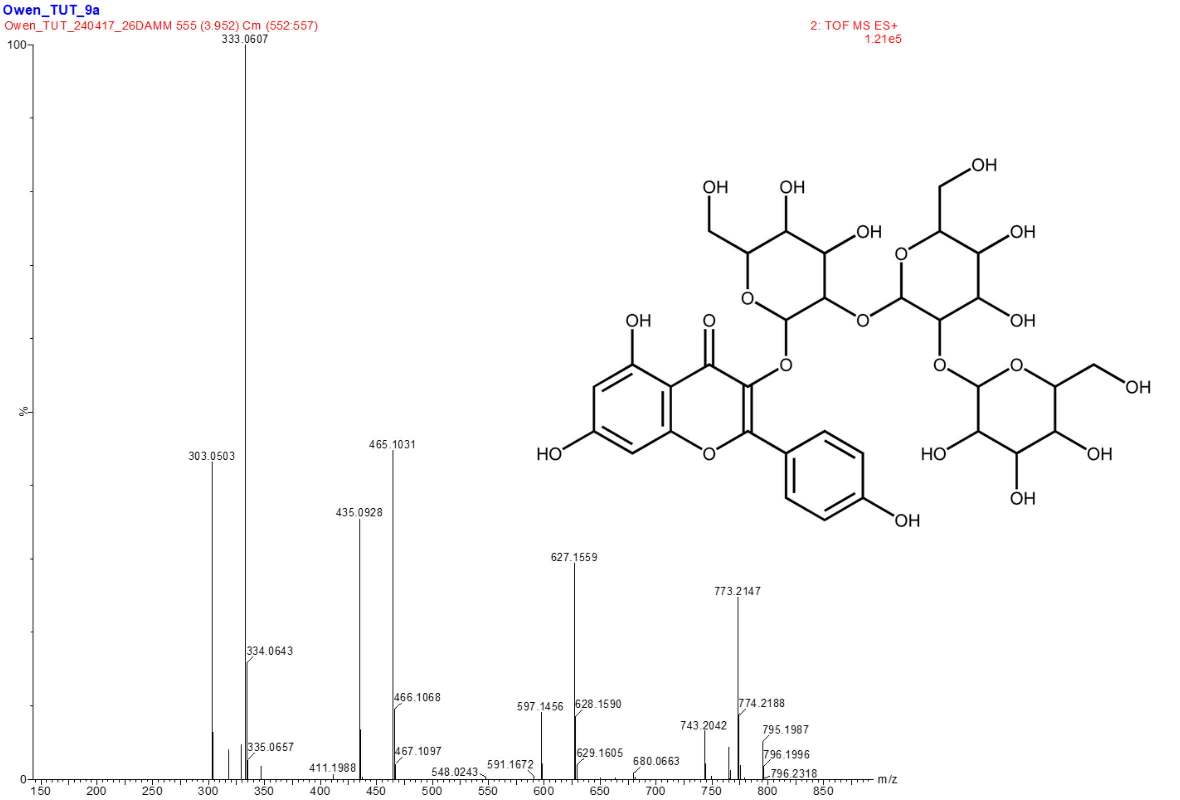

Supplementary Figure S3: MS/MS spectrum of Kaempferol 3-sophorotrioside adjacent to its chemical structure

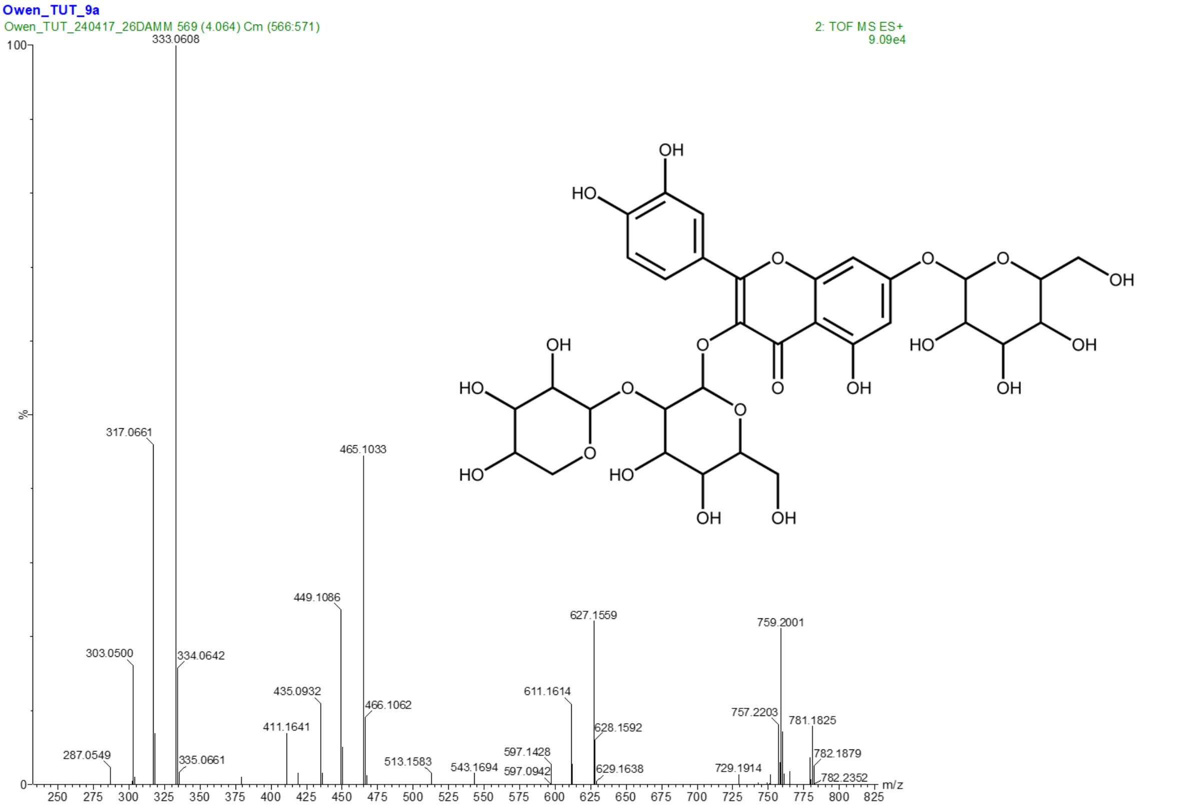

Supplementary Figure S4: MS/MS spectrum of Quercetin 3-sambubioside-7-glucoside adjacent to its chemical structure

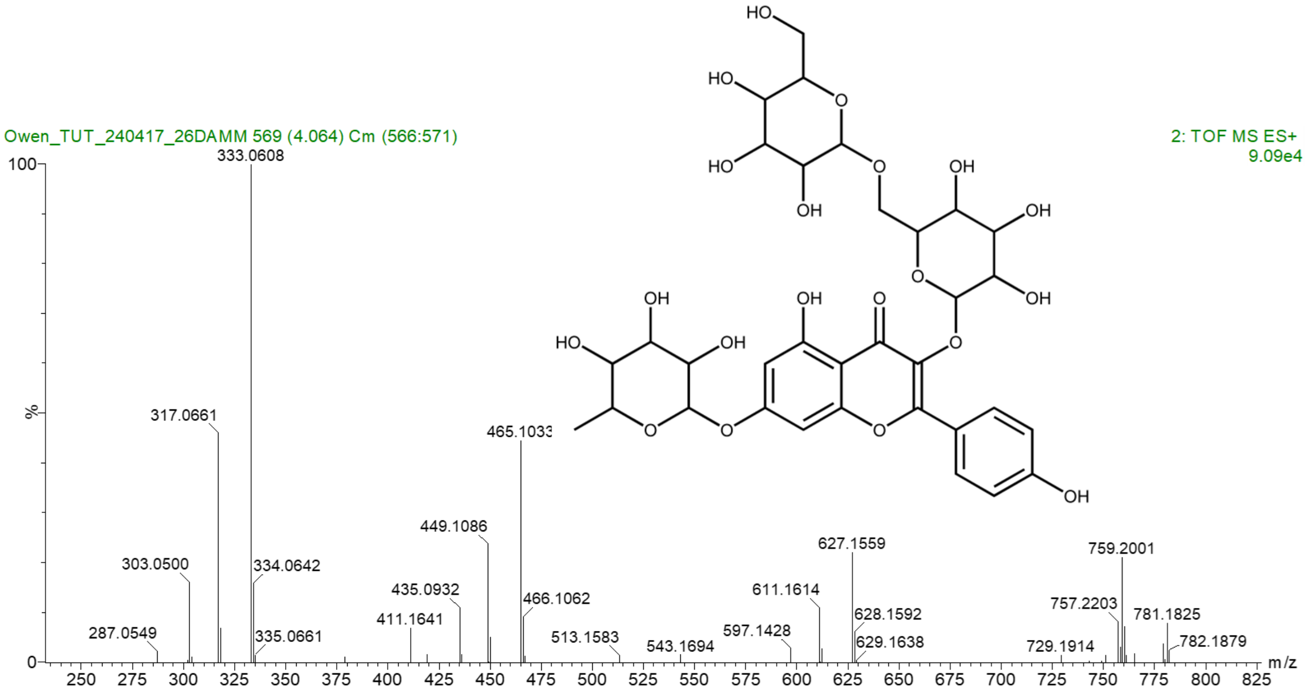

Supplementary Figure S5: MS/MS spectrum of Kaempferol 3-gentiobioside 7-rhamnoside adjacent to its chemical structure

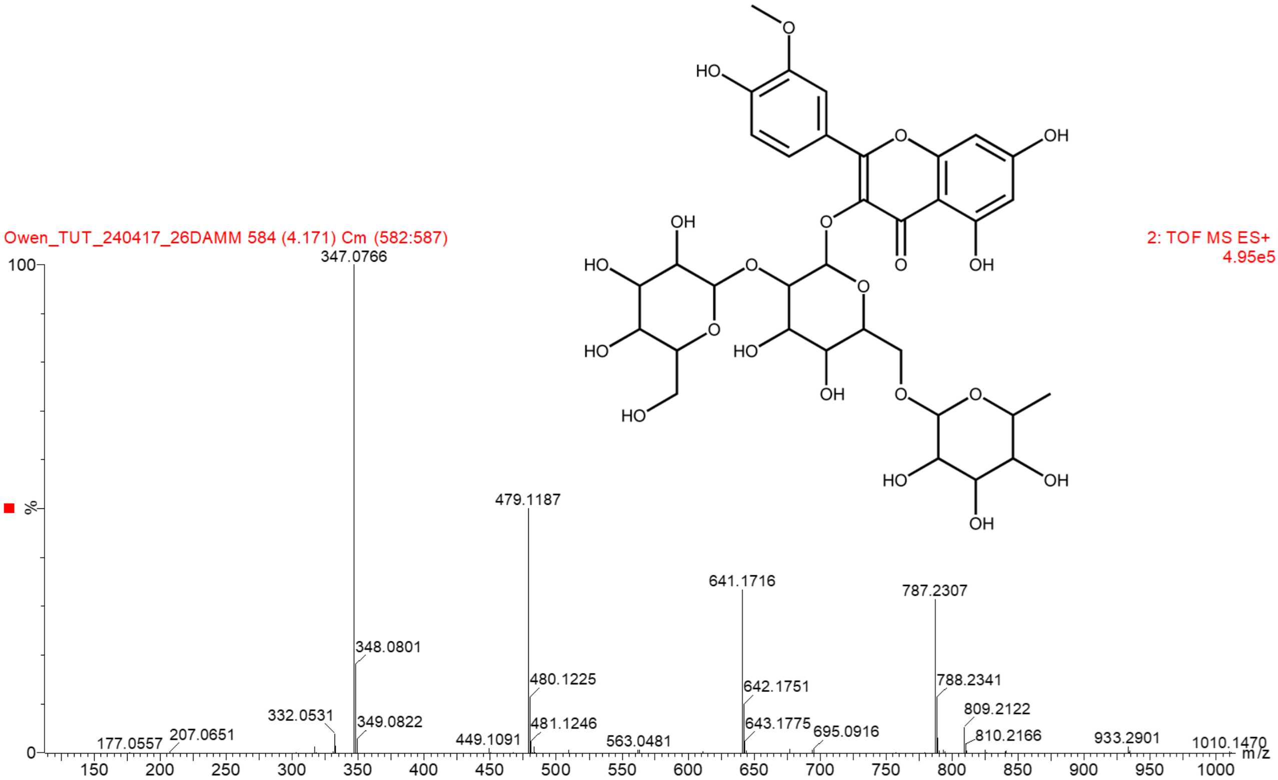

Supplementary Figure S6: MS/MS spectrum of Isorhamnetin 3-O-[b-D-glucopyranosyl-(1->2)-[a-L-rhamnopyranosyl-(1->6)]-b-D-glucopyranoside] adjacent to its chemical structure

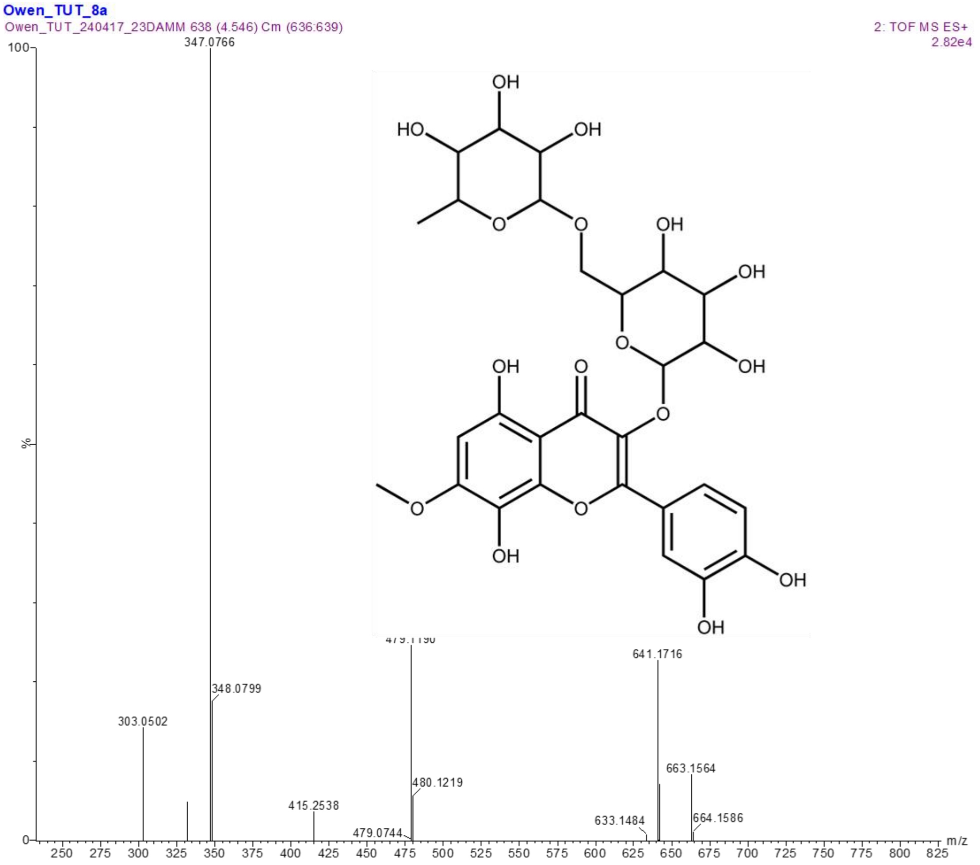

Supplementary Figure S7: MS/MS spectrum of Ranupenin 3-rutinoside and its chemical structure

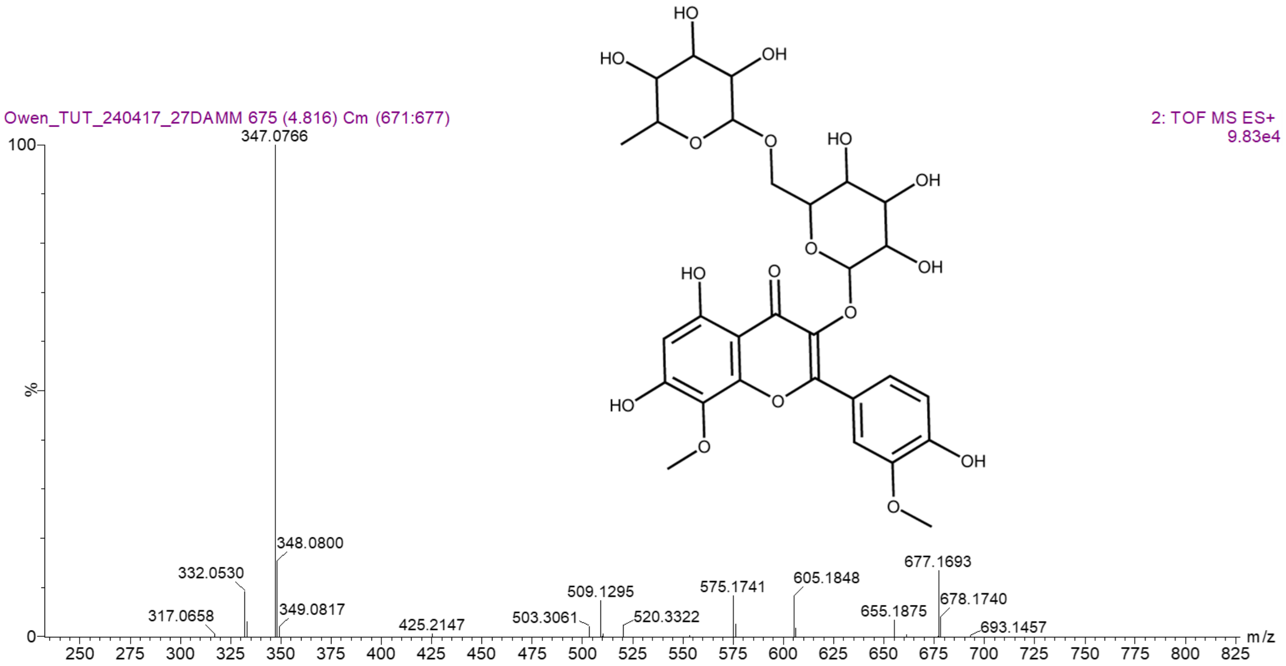

Supplementary Figure S8: MS/MS spectrum of Limocitrin 3-rutinoside with its chemical structure

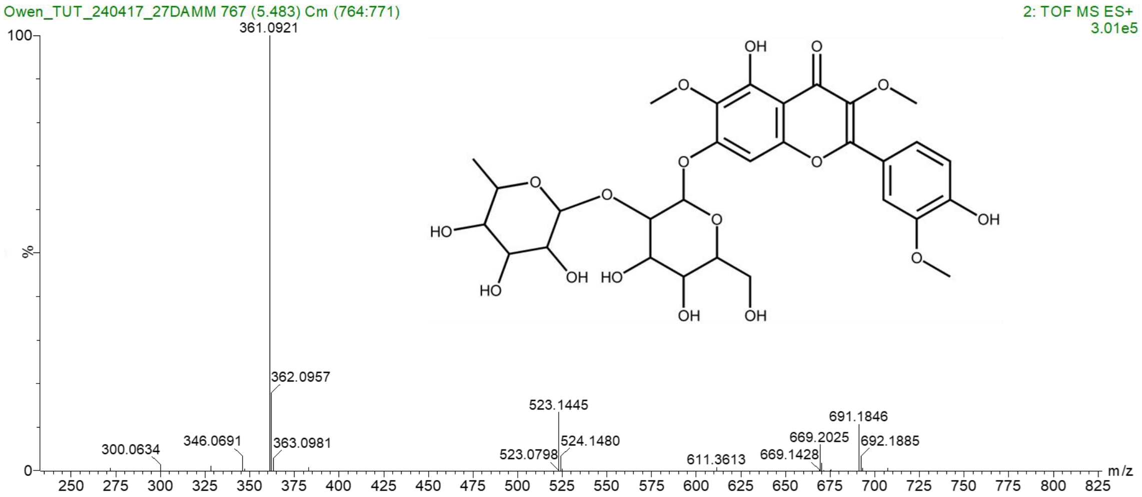

Supplementary Figure S9: MS/MS spectrum of Jaceidin 7-neohesperidoside with its chemical structure
